# Protective Effect of Melatonin on Acute Pancreatitis

**DOI:** 10.1155/2012/173675

**Published:** 2012-04-23

**Authors:** Jolanta Jaworek, Joanna Szklarczyk, Andrzej K. Jaworek, Katarzyna Nawrot-Porąbka, Anna Leja-Szpak, Joanna Bonior, Michalina Kot

**Affiliations:** Department of Medical Physiology, Faculty of Health Sciences, Jagiellonian University, Medical College, Michalowskiego 12, 31-126, Krakow, Poland

## Abstract

Melatonin, a product of the pineal gland, is released from the gut mucosa in response to food ingestion. Specific receptors for melatonin have been detected in many gastrointestinal tissues including the pancreas. Melatonin as well as its precursor, L-tryptophan, attenuates the severity of acute pancreatitis and protects the pancreatic tissue from the damage caused by acute inflammation. The beneficial effect of melatonin on acute pancreatitis, which has been reported in many experimental studies and supported by clinical observations, is related to: (1) enhancement of antioxidant defense of the pancreatic tissue, through direct scavenging of toxic radical oxygen (ROS) and nitrogen (RNS) species, (2) preservation of the activity of antioxidant enzymes; such as superoxide dismutase (SOD), catalase (CAT), or glutathione peroxidase (GPx), (3) the decline of pro-inflammatory cytokine tumor necrosis **α** (TNF**α**) production, accompanied by stimulation of an anti-inflammatory IL-10, (4) improvement of pancreatic blood flow and decrease of neutrophil infiltration, (5) reduction of apoptosis and necrosis in the inflamed pancreatic tissue, (6) increased production of chaperon protein (HSP60), and (7) promotion of regenerative process in the pancreas. *Conclusion*. Endogenous melatonin produced from L-tryptophan could be one of the native mechanisms protecting the pancreas from acute damage and accelerating regeneration of this gland. The beneficial effects of melatonin shown in experimental studies suggest that melatonin ought to be employed in the clinical trials as a supportive therapy in acute pancreatitis and could be used in people at high risk for acute pancreatitis to prevent the development of pancreatic inflammation.

## 1. Melatonin in Pineal Gland and in the Gastrointestinal Tract

More than 50 years ago, Aaron Lerner, a dermatologist from Yale University, discovered melatonin (5-methoxy-N-acetyltryptamine) in the pineal gland. The name of this indoleamine comes from its effect on melanocytes [1]. Melatonin is produced from amino acid precursor; L-tryptophan and its production and release from the pineal gland undergo rhythmic diurnal/nocturnal fluctuations, with the peak at night and lowest level on the light phase [2–4]. 

 Although melatonin has been recognized as the pineal hormone, subsequent studies have shown that melatonin could be synthesized in many tissues and that the gastrointestinal tract appears to be the main source of this substance [5–7]. Two main enzymes involved in the control of melatonin production, *arylalkylamino*-*N-acetyl-serotonin-transferase *(AA-NAT) and *hydroxyindolo-O-methyl-transferase* (HIOMT), have been detected in the gastrointestinal system [8, 9]. In addition, the estimated level of melatonin in the gastrointestinal tract was 400 times higher than that in the pineal gland [10]. In the gut, this indoleamine is produced mainly in the enteroendocrine (EE) cells; however, high concentration of melatonin has also been found in the bile [11–14]. Melatonin is released from the gastrointestinal mucosa in response to ingested food and this process is independent from the light/dark cycle and from the pineal gland [7, 15].

 Previous studies have suggested that melatonin could be produced in the pancreas; the mRNA signal for AA-NAT, an enzyme involved in the synthesis of melatonin from L-tryptophan, has been detected in the isolated rat pancreatic acinar cells [16], and gene expression for HIOMT, another enzyme controlling the above reaction has been, discovered in the human pancreas [17]. Recent studies revealed that melatonin and its receptors are present in the pancreatic gland [18]. Even though melatonin production in the pancreas is independent from the pineal gland, the content of melatonin in the pancreatic tissue undergoes rhythmic diurnal/nocturnal fluctuations [19].

 It is interesting to compare diurnal/nocturnal fluctuations of melatonin in the pancreas, pineal gland, and in the plasma. Nocturnal concentration of melatonin in the pineal gland was about 1600 pg/100 mg, whereas, in the daytime, this concentration was reduced to about 150 pg/100 mg. This corresponds to the plasma levels of melatonin, which reaches 150 pg/mL at night, whereas, throughout the day, it was much lower (60 pg/mL). In the pancreatic tissue, the above-mentioned differences between nocturnal concentration of melatonin and its daily content were less pronounced and achieved 10 and 5 pg/100 mg of tissue, respectively [18].

 Melatonin membrane receptors have been identified in the human endocrine pancreas, and this indoleamine has been proposed as one of the modulators of insulin secretion [20]. The abnormal secretion of melatonin was observed in type 2 diabetes, which may contribute to the pathogenesis of this disease [21–24]. In spite of the presence of melatonin in the pancreas and melatonin receptors in the pancreatic tissue, the role of this substance in the physiological regulation of pancreatic functions is still not complete.

 Melatonin has also been recognized as a potent pancreatic secretagogue. Administration of melatonin or its amino acid precursor, L-tryptophan, to the animals resulted in the spectacular enhancement of pancreatic enzyme secretion accompanied by a significant increase of CCK plasma level [25]. Stimulatory effects of melatonin or L-tryptophan were much stronger following intraduodenal than intraperitoneal administration of investigated substances. The results of experimental studies suggest that melatonin activates cholinergic enteropancreatic reflex to increase pancreatic enzyme secretion [26]. It is very likely that melatonin produced in the gut lumen in response to food ingestion is implicated in the physiological regulation of pancreatic exocrine function.

## 2. Anti-Inflammatory Effects of Melatonin

 The physiological significance of melatonin was the subject of several studies. Because of its rhythmic diurnal/nocturnal fluctuations, melatonin was believed to regulate the circadian rhythms such as hormones release, sleep/wake, and changes of body temperature [2–4, 27]. However, the most interesting property of melatonin appears to be its anti-inflammatory effect. Melatonin has been recognized as a beneficial substance, effectively protecting the tissues from inflammatory damage [16, 17, 28–33]. The favorable effect of melatonin, which has been reported in several studies, depends on two main mechanisms: (1) antioxidant effects of this indole and (2) modulation of immune defense induced by melatonin [34–41]. 

 Melatonin is best known as the scavenger of radical oxygen (ROS) and nitrogen (RNS) species and activator of antioxidant enzymes [29, 42–47]. ROS and RNS are products of mitochondrial metabolism, and, under normal conditions, they are immediately neutralized by natural nonenzymatic scavengers and antioxidant enzymes. Melatonin together with reduced glutathione, vitamins C and E, uric acid, selenium, and creatinine belong to nonenzymatic scavengers [44, 48, 49]. Antioxidant enzymes such as; superoxide dismutase (SOD), catalase (CAT), glutathione peroxydase (GPx), or glutathione reductase (GR) are another line of defense against the noxious effect of ROS and RNS [17, 29, 43–45]. Oxidative stress in acute pancreatitis resulted in excessive production of ROS and RNS leading to the impaired ability of tissue to detoxify above intermediates. ROS and RNS are accumulated in the tissue leading to its damage [50–52]. The harmful effects of ROS and RNS in acute pancreatitis have been confirmed in previous studies [53, 54]. Melatonin, which is a highly lipophilic substance, penetrates inside the cells to maintain antioxidant enzymes activities, to keep the mitochondria from oxidative injury, and to prevent lipid membranes from peroxidation [55].

 Recently melatonin has been shown to trigger signal transduction pathways leading to the activation of antioxidant enzymes and to the reduction of inflammatory mediators in the pancreas [56]. In acute pancreatitis, melatonin was demonstrated to inhibit nuclear binding of NF-*κ*B, the transcription factor, which controls the expression of genes involved in immunity and inflammation, production of prostaglandins, cytokines, cell adhesion molecules, nitric oxide (NO), and inhibitors of apoptosis [57, 58]. Melatonin has been demonstrated to reduce gene expression and synthesis of proinflammatory cytokine; tumor necrosis factor *α* (TNF*α*), proinflammatory interleukins; IL-1*β*, IL-6, IL-8, and prostaglandins [56, 59, 60]. In addition, melatonin was also reported to modulate the processes of apoptosis and necrosis, to stimulate the production of vascular endothelial growth factor (VEGF), and to activate the process of angiogenesis [32, 61, 62]. All of the above effects could be related to the inhibition of NF-*κ*B by melatonin [56].

 Beside the reduction of the above proinflammatory molecular pathway, melatonin enhanced expression of nuclear factor erythroid 2-related factor (Nrf2), which activates the signal transduction lane of antioxidant genes [56]. Since Nrf2 is critically involved in the induction of antioxidant enzymes such as SOD, CAT, GPx, GST, GR [63] and melatonin is known to activate above enzymes, it is quite possible that the antioxidative effect of melatonin is mediated by Nrf2.

 Melatonin binds to the specific G-protein-coupled receptors: MT_1_R, MT_2_R, MT_3_R, which have been identified in many tissues [64]. Perhaps melatonin exerts its effects also through intracellular orphan receptors (ROR/RZR), which have been detected in the nucleus, cytoplasm, and mitochondria, but physiological significance of these receptors remains unclear [64, 65]. It is important to emphasize that melatonin could very easily cross the cell membranes because it is highly lipophilic, and on this way melatonin could exert its biological receptor-independent effects [66].

 Production of melatonin is decreased in older individuals, and it is believed that the reduction of melatonin level contributes to the process of aging [67]. Application of this substance reverses some of degenerative processes related to old age, and, for this reason, melatonin was suggested to be the “hormone of youth” [3, 31, 67].

## 3. Beneficial Effects of Melatonin on Acute Pancreatitis

**Figure 1:**
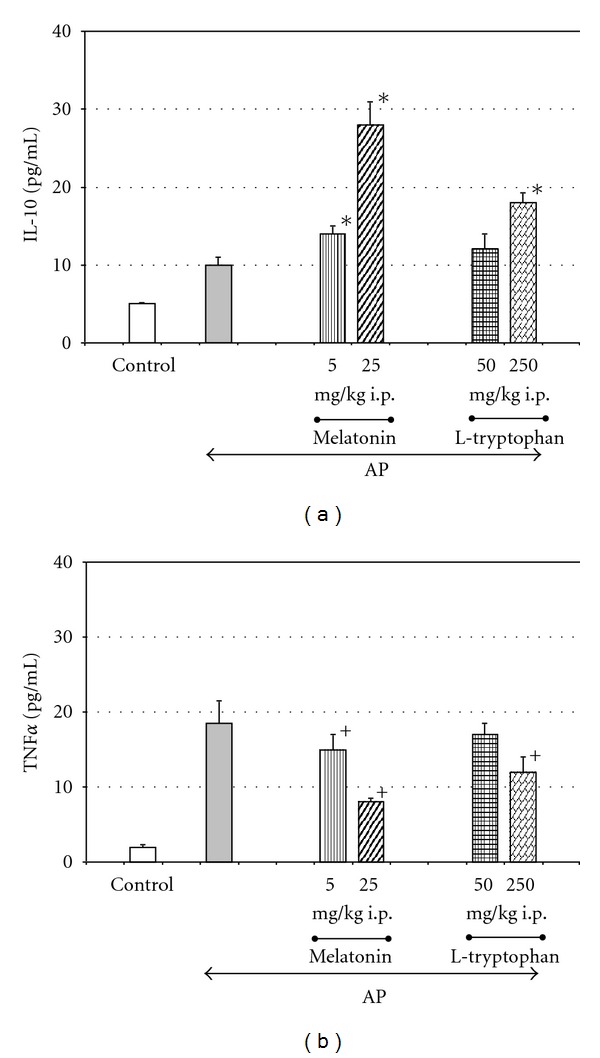
Effect of melatonin on plasma levels of interleukin 10 (IL-10) and tumor necrosis factor *α* (TNF *α*) in the rats with acute pancreatitis (AP). Melatonin or its precursor, L-tryptophan, was given to the rats 30 min prior the induction of acute pancreatitis produced by caerulein overstimulation (5 *μ*g/kg-h × 5 h). Control-intact rats. Means ± SEM from the separate experiments, each performed on 8–10 rats. Asterisk indicates significant increase above the value detected in AP rats alone. Cross indicates significant decreases below the value detected in AP rats alone.

 Experimental studies have shown that application of melatonin significantly attenuated the development of acute pancreatitis and protected pancreatic tissue against the damage caused by acute inflammation [16, 17, 59–61, 68–75]. In the rats pretreated with melatonin prior to the induction of acute pancreatitis, the morphological signs of inflammation such as edema, leukocyte infiltration, and cell vacuolization were dramatically reduced [16, 60, 70–73]. Also other parameters of acute pancreatitis severity such as blood levels of amylase or lipase were significantly diminished in the animals pretreated with melatonin, as compared to the rats with acute pancreatitis alone [16, 17, 59, 68, 70–75]. The beneficial effect of this indoleamine on acute pancreatitis was also manifested by the dose-dependent reduction of proinflammatory cytokine TNF*α* blood level, accompanied by a marked rise of anti-inflammatory interleukin 10 (IL-10) in the animals subjected to acute pancreatitis and pretreated with melatonin [16, 69, 70] (Figure 1). 

**Figure 2:**
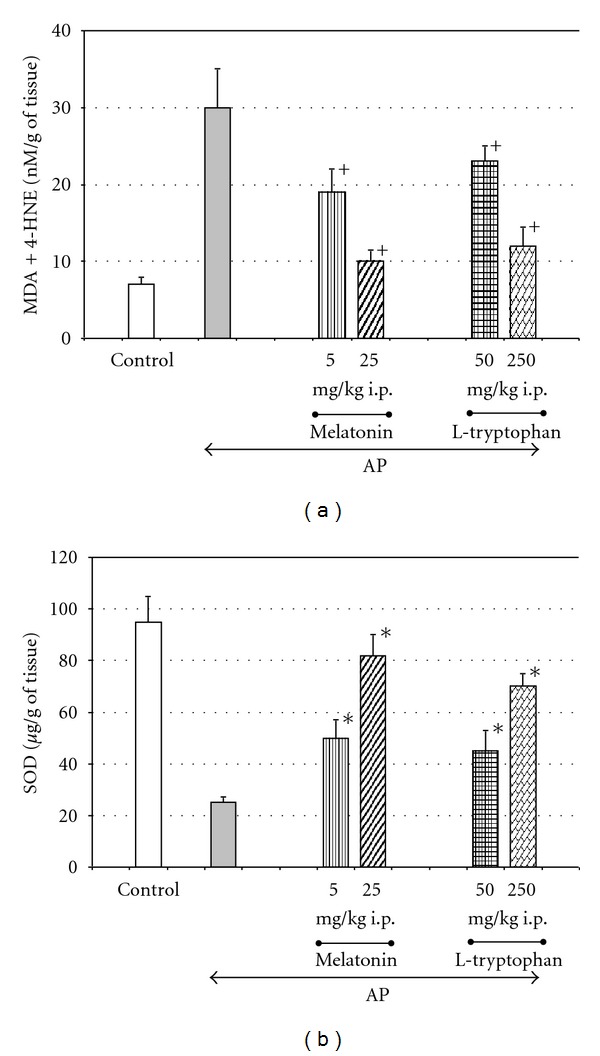
Effect of intraperitoneal (i.p.) application of melatonin or its precursor, L-tryptophan, on concentrations of antioxidative enzyme superoxide dismutase (SOD) and lipid peroxidation products (MDA + 4-HNE) in the pancreatic tissue taken from rats with acute pancreatitis (AP). Melatonin or its precursor, L-tryptophan, was applied as explained on Figure 1. Control—intact rats. Means ± SEM from the separate experiments, each performed on 8–10 rats. Asterisk indicates significant increase above the value detected in AP rats alone. Cross indicates significant decreases below the value detected in AP rats alone.

 Melatonin is able to diminish the generation of ROS in the pancreatic tissue, as was demonstrated by the reduced amount of lipid peroxidation products: MDA + 4HNE in the pancreas of animals with acute pancreatitis pretreated with this indoleamine [16, 17, 68, 70–73, 75]. In addition, the application of the mentioned protective substance resulted in the significant and dose-dependent increase of antioxidant enzyme (SOD) activity in the pancreatic tissue taken from the rats with acute pancreatitis [17, 61, 72–74] (Figure 2).

 The protective action of melatonin on acute pancreatitis was confirmed in several studies, using different models of experimental pancreatitis. Melatonin attenuated acute pancreatitis severity and diminished harmful effects of acute inflammation induced by L-arginine [72], ischemia/reperfusion, or caerulein overstimulation [16, 59, 61, 68, 70, 74, 76]. Melatonin protected the pancreas against acute pancreatitis caused by taurocholic acid [60] or by obstruction of pancreatic duct [75]. However, in the model of necrotizing pancreatitis induced by glycodeoxycholic acid melatonin appears less effective, because increased serum amylase level and high mortality rate of experimental animals was unaffected by this indoleamine [77].

 It is worth remembering that this substance was also found to promote the regeneration of pancreatic tissue following the damage caused by acute pancreatitis. Treatment with melatonin improves the rate of DNA synthesis, as well as pancreatic enzyme content in the rats with arginine-induced pancreatitis [76].

**Figure 3:**
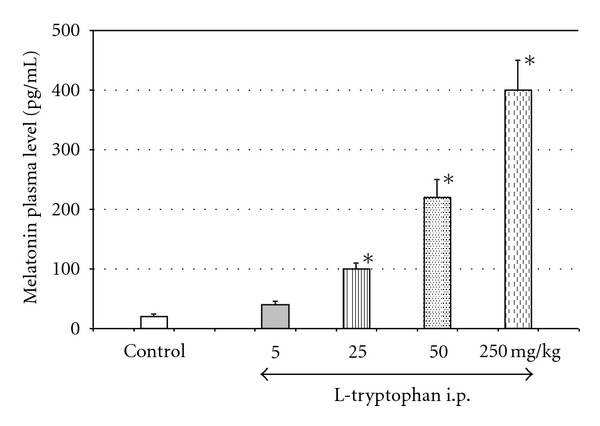
Plasma level of melatonin in response to intraperitoneal (i.p.) administration of increasing doses of L-tryptophan. Melatonin plasma level was measured by RIA. Means ± SEM from the separate experiments, each performed on 8–10 rats. Asterisk indicates significant increase above the control value.

 Studies on melatonin revealed that not only melatonin but also its amino acid precursor, L-tryptophan, is able to attenuate pancreatic tissue damage caused by acute inflammation and to reduce lipid peroxidation in two models of acute pancreatitis: caerulein-induced and ischemia/reperfusion pancreatitis [16]. It is likely that the protective effect of L-tryptophan on acute pancreatitis was dependent on the conversion of this amino acid into melatonin, because intraduodenal administration of L-tryptophan resulted in the significant and dose-dependent increase of plasma melatonin level (Figure 3). These results lead to the conclusion that endogenous melatonin, which is produced from L-tryptophan effectively protects the pancreas from the damage caused by acute inflammation [16].

 Application of L-tryptophan at doses of 25 or 50 mg/kg raised the plasma level of melatonin up to 100 or 220 pg/mL, respectively. It is important to underline that pretreatment of the animals with the above doses of L-tryptophan significantly attenuates the inflammatory process in the pancreas [16]. Since normal blood level of melatonin fluctuates from 50 pg/mL (during the light phase) to about 160 pg/mL (at night) [18], it could be assumed that melatonin at physiological concentrations is able to protect the pancreas against acute inflammatory damage.

 This observation indicates that endogenous melatonin could be one of the physiological protectors of the pancreas. This notion is supported by the study showing that blockade of the melatonin receptor aggravated pancreatic damage caused by caerulein overstimulation. In the rats subjected to acute pancreatitis and pretreated with melatonin MT1/2 receptor antagonist, luzindole, the histological and biochemical manifestations of pancreatitis were significantly higher than in the group with acute pancreatitis alone [70].

 Recent observation from humans with acute pancreatitis supported and reinforced this hypothesis. It was observed that melatonin serum level, measured in the first 24 hours after the onset of acute pancreatitis, negatively correlated with the severity of this illness. In the patients with mild pancreatitis, serum level of melatonin was markedly higher than in these with severe form of this disease [78]. This study presents additional evidence that melatonin could be one of the natural pancreatic protectors and that high blood level of this indoleamine has a protective value against acute pancreatic inflammation.

**Figure 4:**
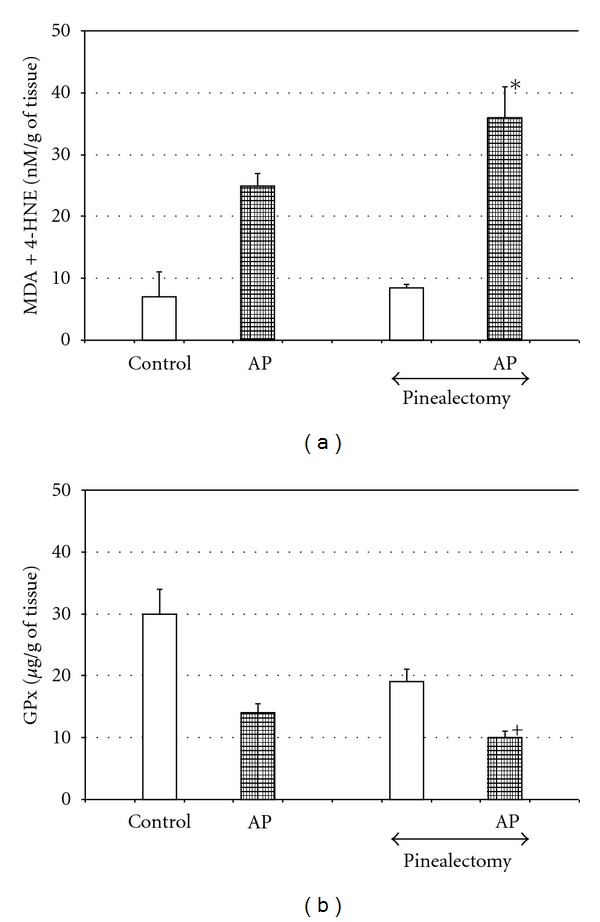
Effect of pinealectomy on pancreatic contents of glutathione peroxidase (GPx) and lipid peroxidation products (MDA + 4-HNE) in the pancreatic tissue of rats with acute pancreatitis. Control—intact rats. Means ± SEM from the separate experiments, each performed on 8–10 rats. Asterisk indicates significant increase above the value detected in AP with intact pineal gland. Cross indicates significant decrease below the value detected in AP with intact pineal gland.

 A considerable amount of melatonin is produced in the gastrointestinal system in response to food ingestion, and this melatonin is absorbed into the blood stream and represents the daily pool of this indoleamine, whereas nocturnal level of melatonin depends on its synthesis in the pineal gland [5, 7, 12, 33]. The possible involvement of this pineal melatonin in the pancreatic protection should be taken into consideration, and, to solve this problem, subsequent experiments have been performed. It has been observed that acute pancreatitis induced in the dark phase was more severe than during the day [74]. Because nocturnal melatonin plasma level depends on the production and release of this substance from the pineal gland, it is possible that pineal melatonin takes part in the protection of the pancreas against acute inflammation. A recent study on rats with removed pineal glands give further evidence for this hypothesis. As we have observed, acute pancreatitis was much more severe in pinealectomized animals, than in those with intact pineal glands. This was manifested by significant decrease of an antioxidant enzyme GPx in the pancreas of pinealectomized rats subjected to acute pancreatitis, as compared to the animals with intact pineal glands (Figure 4). Application of melatonin to the rats deprived of the pineal gland and subjected to acute pancreatitis significantly reduced pancreatic tissue lesions and attenuated the course of acute inflammation [75].

 The results of experimental studies and clinical observations indicate that several mechanisms are involved in the beneficial effect of melatonin on acute pancreatitis.

### 3.1. Antioxidative Mechanism

 Melatonin as an effective scavenger of free radicals is able to neutralize above toxic products directly [16, 17, 68, 70–73]. In addition, this indoleamine could activate the antioxidant enzymes such as SOD, CAT, GPx, and GSH and protects them from inactivation by reactive intermediates. Thus, melatonin could improve the oxidative status of the pancreatic tissue indirectly [16, 61, 70, 71, 73–75].

### 3.2. Modulation of the Immune System

Melatonin is able to affect the immune system and to strengthen the immune defense. This substance has been demonstrated to inhibit neutrophile infiltration [16, 70–74], to decrease myeloperoxidase (MPO) activity [56], and to diminish the prostaglandin generation [59] in the inflamed pancreas. Recently, melatonin has been shown to reduce mRNA expression of many proinflammatory cytokines such as IL-1 *β*, IL-6, IL-8, and TNF*α* in the pancreatic tissue subjected to acute inflammation [56]. The inhibitory effect of melatonin on proinflammatory cytokine production has been confirmed by marked reduction of the blood level of TNF*α* in the rats with acute pancreatitis pretreated with this indoleamine [16, 69, 70]. On the contrary; anti-inflammatory IL-10 was increased in these animals [16]. Melatonin enhanced the expression of nuclear factor erythroid 2-related factor (Nrf2) and diminished the nuclear binding of NF-*κ*B, and it is likely that above effects could be involved in the curtailing of acute pancreatitis by melatonin [56].

### 3.3. Improvement of Pancreatic Blood Flow

Melatonin has been demonstrated to increase the blood flow and to remove the toxic substances from pancreatic tissue [16, 69, 70, 76, 79].

### 3.4. Effect on Apoptosis

Melatonin is able to reduce processes of apoptosis and necrosis in the pancreas [61]. However in the tumor cells, this substance promotes apoptosis maintaining the viability of normal pancreatic units [80].

### 3.5. Stimulation of Heat Shock Protein (HSP)

HSPs are known to protect the cell compartment against the damage. Production of these proteins is augmented in response to high temperature, oxidative stress, or inflammation [81]. Melatonin has been reported to increase mRNA signal for HSP60 in pancreatic acinar cell line AR42J [82]. It could be expected that melatonin works to save acinar cells from acute damage through the stimulation of HSP production. As was observed, melatonin prevented these cells from mitochondrial and nuclear damage caused by acute pancreatitis, reduced the dilatation of endoplasmatic reticulum and Golgi apparatus, and diminish formation of autophagosomes [72].

### 3.6. Promotion of Pancreatic Regeneration

It should be emphasized that administration of melatonin following the induction of acute pancreatitis not only reduced the severity of inflammation, but also promotes the spontaneous regeneration process of the pancreatic tissue. This was manifested by an increased rate of DNA and protein synthesis and supported by histological examination [77].

 The results of previous experimental studies and clinical test indicate that melatonin should be employed in clinical trials as a supportive agent for the treatment of patients with acute pancreatitis. Melatonin has been used previously as part of composed therapy in patients with tumors and neurological diseases [82–84]. In patients with cancer melatonin significantly decreased thrombocytopenia, asthenia, neuro- and cardiotoxicity induced by chemotherapeutic treatment [82]. It is important to emphasize that the use of this indoleamine is safe, it has been reported that melatonin given at doses of 20 mg/day for several weeks in patients with dyskinesia or with sleep disturbance did not produce any side effects [84, 85]. It was suggested that melatonin at doses as high as 50–100 mg/day could be applied for treatment of insomnia and depression [85]. Regarding the beneficial effects and safety of melatonin use, this substance could also be introduced as a component of early jejunal feeding in patients with acute pancreatitis.

## 4. Conclusion

Endogenous melatonin could be one of the native mechanisms protecting the pancreas from acute damage and accelerating regeneration of this gland. The beneficial effects of melatonin shown in experimental studies suggest that melatonin ought to be employed in the clinical trials as a adjuvant therapy in acute pancreatitis.
